# The Importance of Computer Science for Public Health Training: An Opportunity and Call to Action

**DOI:** 10.2196/publichealth.5018

**Published:** 2016-03-14

**Authors:** Sarah Kunkle, Gillian Christie, Derek Yach, Abdulrahman M El-Sayed

**Affiliations:** ^1^ The Vitality Group New York, NY United States; ^2^ Detroit Health Department City of Detroit Detroit, MI United States

**Keywords:** digital health, public health, machine learning, computer science, health technology, chronic disease

## Abstract

A century ago, the Welch-Rose Report established a public health education system in the United States. Since then, the system has evolved to address emerging health needs and integrate new technologies. Today, personalized health technologies generate large amounts of data. Emerging computer science techniques, such as machine learning, present an opportunity to extract insights from these data that could help identify high-risk individuals and tailor health interventions and recommendations. As these technologies play a larger role in health promotion, collaboration between the public health and technology communities will become the norm. Offering public health trainees coursework in computer science alongside traditional public health disciplines will facilitate this evolution, improving public health’s capacity to harness these technologies to improve population health.

## Introduction

In 1915, the Rockefeller Foundation published a report by William Welch and Wickliffe Rose to delineate a knowledge base for public health practice in the United States and to design an educational system accordingly. While compiling this report, Welch, Rose, and other stakeholders struggled with the multidisciplinary nature of the field. Most professions are defined by a common disciplinary focus, but public health combines diverse disciplines to achieve a common goal [1]. Distinct from medicine and health care, public health focuses on promoting health and preventing disease at the population level. While a deep knowledge of biological and life sciences forms the core of medical training, public health requires a more comprehensive set of skills, including biology and life sciences, social sciences, public policy, and statistical reasoning [2].

The Council on Education for Public Health (CEPH), an independent agency recognized by the US Department of Education to accredit public health schools and programs, emphasizes five core areas that constitute the “intellectual framework” for public health professionals: biostatistics, epidemiology, environmental health sciences, health services administration, and social and behavioral sciences [3]. One of the CEPH’s three objectives is to encourage—through periodic review, consultation, research, publications, and other means—making improvements in the quality of education for public health [4].

Since the formation of the CEPH in 1974, several reports have assessed the state of public health and made recommendations for public health education. In 1988, a US Institute of Medicine (now the National Academy of Medicine) report on the future of public health called for a greater emphasis on public health practice and relationships with academic disciplines outside of public health, including business administration and departments of physical, biological, and social sciences [5]. Following up on that report, in 2002 the Institute of Medicine again highlighted the need for public health schools to cross traditional boundaries and provide transdisciplinary training. This report specifically emphasized the need for training in computer skills and information technology [6]. Echoing these sentiments, *The Lancet* Commission on the Education of Health Professionals for the 21st Century also stressed the need for the next generation of learners to “discriminate vast amounts of information and extract and synthesize knowledge that is necessary for clinical and population-based decision making” [7].

Many public health programs now offer specialization in public health informatics—the systematic application of information and computer science and technology to public health practice and research [8,9]. Nonetheless, curricula have rarely kept up with the data management and analytic requirements to understand the implications of new technologies [10]. One example of this is disease surveillance—a key responsibility of public health. Advances in information technology have spurred an evolution in our capacity to collect crucial information quickly, remotely, reliably, and cheaply. These technologies allow for the continuous real-time collection and analysis of health-related data. Both Google Search data and Twitter data have provided insights into disease surveillance and other “digital epidemiology” research questions [11-13].

## The Digital and Mobile Health Revolution

Over the last few decades, the digital revolution has fueled technological progress and innovation. It is becoming clear that mobile devices will play a growing role in that process [14]. Smartphone penetration has surpassed that of personal computers, with estimates suggesting that usage will exceed 6 billion by 2020 [15]. With increases in smartphone usage, mobile phone apps have become a ubiquitous presence in users’ lives; most users report using at least 20 apps on their devices [16].

Health apps are particularly popular. A 2014 analysis estimated that there are over 100,000 health, fitness, and medical mobile apps, with the majority focusing on preventive areas such as healthy living, diet and exercise, addiction, stress, relaxation, and sleep [16]. Along with the growing presence of wearable technologies (eg, fitness trackers and smartwatches), these apps are contributing to a surge in the availability of health-related data. These apps collect tremendous information flows, in real time, and have the capacity to interact with the user, enabling changes in user behavior in response to user data.

## Computation and Public Health: Machine Learning as an Example

One example of the potential for computational techniques to improve public health is machine learning. This methodological approach has emerged as a means of making sense of increasingly complex, high-volume big data such as those emerging from apps. Arthur Samuel, a machine learning pioneer, described this domain as the “field of study that gives computers the ability to learn without being explicitly programmed” [17]. Machine learning includes many different methods—regression, decision trees, neural networks, clustering, network analysis—that are more broadly categorized as either supervised or unsupervised learning. Although the field has existed for over half a century, recent progress has allowed for the development of real-world applications, including Google News clustering, Amazon product recommendations, and Facebook photo recognition. Recognizing the demand for machine learning expertise, trainees are flocking to the field; a graduate-level machine learning course is one of the most popular courses at Stanford University [18].

With the emergence of big data, machine learning is increasingly being used in real-world applications that are transforming industries. In 2013, IBM declared that the intersection of cloud computing, big data analytics, and learning technologies would usher in “a new era of cognitive systems where machines will learn, reason and engage with us in a more natural and personalized way” [19]. Large technology companies such as Amazon, Facebook, Google, IBM, and Microsoft have been at the forefront of this movement with investments in machine learning resources (including academic talent). Many smaller startups are also using these methods across a variety of sectors and receiving funding from investors [20]. In 2014, investors put US $309 million into artificial intelligence and machine learning startups across more than 40 deals [21]. Common applications of machine learning include Web search, spam filters, recommender systems, ad placement, credit scoring, and fraud detection [22].

Furthermore, an increasing number of health care stakeholders are recognizing that human-machine collaboration is critical for the development of cost-effective and potentially cost-saving solutions. Google, IBM, and Microsoft have partnered with a variety of health care organizations to implement machine learning solutions for complex problems including medication adherence, cancer treatment, and claims reimbursement. For example, Memorial Sloan Kettering Cancer Center is using IBM Watson Analytics’s cognitive computing technologies to provide oncologists and patients with tailored treatment options informed by clinical evidence and The Center’s highly specialized expertise. Google is working with Stanford University to investigate how machine learning can transform drug discovery by using data from a variety of sources to more accurately identify which chemical compounds could effectively treat a variety of diseases [23].

In the context of public health, computational methods such as machine learning could be used for both predictive and explanatory modeling, that is, identifying which individuals will benefit from an intervention, and better understanding the relationship between different exposures and health outcomes. In the realm of predictive modeling, machine learning could integrate data from a diverse set of sources—electronic health records, genomic sequencing, claims data, mobile sensors, and even social media—to better predict individuals at high risk for specific health conditions. Continually incorporating new data with minimal supervision will likely reduce the time and costs typically associated with building these insights. Once individuals have been identified, interventions and recommendations can be tailored based on personal preferences and feedback. Machine learning allows algorithms to continuously update so they become smarter and more personalized the more they are used. This data-driven approach is an improvement over traditional approaches in which individuals are stratified according to characteristics such as age, sex, and biomarkers to predict risk and recommend interventions.

The promise of machine learning approaches is beginning to be realized. Several technologies in development and in the market are using machine learning methods in concert with behavioral and biometric data to generate personalized suggestions that promote healthier lifestyles without any human involvement [24,25]. Although the literature on efficacy is limited, a recent study of a health-tracking app provided preliminary evidence on machine learning as a tool for behavior change [26]. The app automatically translates behavioral data into personalized suggestions that promote healthier lifestyle without any human involvement. Participants in the experimental group (those who received the app’s personalized suggestions) walked significantly more and rated the suggestions more positively compared with the control group that received nonpersonalized suggestions from professionals. Although the sample size was relatively small and the time period relatively short, these results provide an optimistic outlook for machine learning and health.

Machine learning also has important implications for explanatory modeling and new insights into causality. While randomized controlled trials and experimental data are considered the criterion standard in epidemiology for causal inference, they are often criticized for a lack of external validity [27,28]. What works in a controlled research setting may not translate to an effective solution in practice. As data become increasingly complex, machine learning could help uncover patterns and identify trends, ultimately improving existing explanatory models and generating new causal hypotheses [29].

Although machine learning methods present an opportunity for public health, there are challenges and limitations to consider. Given that these methods generally use a diverse set of data in addition to traditional medical information, there are many concerns relating to data privacy. While the US Health Insurance Portability and Accountability Act protects medical information, existing laws in the United States do not cover data generated by most personalized health technologies. Special consideration must also be given to health inequities because innovative technologies often favor younger and affluent individuals over older, high-risk, and marginalized populations. These novel methods will also inevitably create tension between relying on algorithms and on human recommendations—the true potential of these technologies is their ability to augment rather than replace human expertise.

## A Call to Action for Public Health Training

Big data, machine learning, and other computational techniques have the potential to provide insights into a broad set of public health topics including disease treatment, surveillance, and prevention. Chronic diseases, such as heart disease, stroke, cancer, diabetes, obesity, and arthritis, are the leading causes of death and disability in the United States and in much of the world. Motivating behavioral change related to physical activity, nutrition, tobacco, alcohol use, medication adherence, and mental health could alleviate a substantial portion of the chronic disease burden [30]. Personalized health technologies, specifically those incorporating machine learning, have shown promise in driving behavior change in these areas. If public health practitioners are serious about their commitment to disease prevention, they should follow the lead of health care and other industries in embracing big data and adopting machine learning methods.

A significant constraint in realizing public health value from big data, however, is a shortage of talent at the nexus between public health and computer science. Leading voices, including the Institute of Medicine, the US Centers for Disease Control and Prevention, and *The Lancet* have called attention to the need for information technology skills and have recommended public health curricula changes [6,7,10]. Although many public health programs offer statistical programming courses in SAS and STATA, for example, curricula generally do not include deeper computer programming skills. Some programs have options for specialized training in public health informatics, but gaps in skills and knowledge persist. Computer science disciplines have extended their focus to health, but public health schools have yet to fully embrace computer science. However, the incorporation of computer science into public health training is perhaps more critical than the adoption of public health as a focus for computer science: the role of well-trained public health professionals is essential to foster dialogue on important issues such as the methodological limitations and ethical implications of big data for health.

Public health schools have a history of collaboration and formal engagement with other fields, including medicine, law, nursing, social work, and business [31]. As formalized public health education in the United States celebrates its 100th anniversary, it is time to extend this collaboration to computer science and technology in order to more effectively and efficiently address today’s pressing public health problems.

## Abbreviations

CEPH: Council on Education for Public Health
